# SMS-text messaging for collecting outcome measures after acute stroke

**DOI:** 10.3389/fdgth.2023.1043806

**Published:** 2023-02-23

**Authors:** Julie A. DiCarlo, Kimberly S. Erler, Marina Petrilli, Kristi Emerson, Perman Gochyyev, Lee H. Schwamm, David J. Lin

**Affiliations:** ^1^ Department of Neurology, Massachusetts General Hospital, Boston, MA, United States; ^2^ School of Health and Rehabilitation Sciences, MGH Institute of Health Professions, Boston, MA, United States; ^3^ Digital Enterprise Service, Mass General Brigham, Somerville, MA, United States

**Keywords:** stroke, digital health, SMS-text, outcomes, recovery

## Abstract

**Introduction:**

Traditional methods for obtaining outcomes for patients after acute stroke are resource-intensive. This study aimed to examine the feasibility, reliability, cost, and acceptability of collecting outcomes after acute stroke with a short message service (SMS)-text messaging program.

**Methods:**

Patients were enrolled in an SMS-text messaging program at acute stroke hospitalization discharge. Participants were prompted to complete assessments including the modified Rankin scale (mRS) and Patient-Reported Outcomes Measurement (PROM) Information System Global-10 at 30, 60, and 90 days postdischarge *via* SMS-text. Agreement and cost of SMS-text data collection were compared to those obtained from traditional follow-up methods (*via* phone or in the clinic). Participant satisfaction was surveyed upon program conclusion.

**Results:**

Of the 350 patients who agreed to receive SMS texts, 40.5% responded to one or more assessments. Assessment responders were more likely to have English listed as their preferred language (*p* = 0.009), have a shorter length of hospital stay (*p* = 0.01), lower NIH stroke scale upon admission (*p* < 0.001), and be discharged home (*p* < 0.001) as compared to nonresponders. Weighted Cohen’s kappa revealed that the agreement between SMS texting and traditional methods was almost perfect for dichotomized (good vs. poor) (*κ *= 0.8) and ordinal levels of the mRS score (*κ *= 0.8). Polychoric correlations revealed a significant association for PROM scores (ρ = 0.4, *p* < 0.01 and ρ = 0.4, *p* < 0.01). A cost equation showed that gathering outcomes *via* SMS texting would be less costly than phone follow-up for cohorts with more than 181 patients. Nearly all participants (91%) found the program acceptable and not burdensome (94%), and most (53%) felt it was helpful. Poststroke outcome data collection *via* SMS texting is feasible, reliable, low-cost, and acceptable. Reliability was higher for functional outcomes as compared to PROMs.

**Conclusions:**

While further validation is required, our findings suggest that SMS texting is a feasible method for gathering outcomes after stroke at scale to evaluate the efficacy of acute stroke treatments.

## Introduction

Stroke is a leading cause of acquired adult disability worldwide (1). Recent substantial advances in acute stroke treatments (2, 3) and novel approaches to stroke rehabilitation (4, 5) have resulted in significant improvements in poststroke outcomes. To systematically evaluate the real-world benefit of such interventions, it is essential to reliably collect outcomes for patients after acute stroke discharge.

Current approaches to outcomes data collection face many logistical barriers. Follow-up care, during which outcomes are traditionally collected, requires patients to return to specialized stroke centers and can be time-intensive, cost-prohibitive, and burdensome, relying on interaction with trained healthcare providers (6, 7). Phone calls to stroke patients to assess outcomes are also time-consuming and require dedicated and trained staff.

In recent years, there has been rapid adoption of digital and telehealth approaches in clinical care (8–10). Since mobile phones are one of the most popular forms of digital interaction (11) and are ubiquitous even among diverse demographic groups (11, 12), there is the potential to utilize short message service (SMS) texting for gathering assessments after stroke. SMS-texting programs have been used in a range of health conditions (13–15) for varying utilities, including intervention (16, 17), adherence (13), and data collection (18). Although app-based collection of outcomes after stroke has been explored (19), the feasibility of using SMS texting has not yet been examined in stroke. This study aimed to examine the feasibility, reliability, cost, and acceptability of an SMS-texting approach to gather health outcomes in the first 90 days after acute stroke.

## Materials and methods

Our health system has articulated a goal of collecting functional outcomes after acute stroke discharge on all patients but has lacked the resources to accomplish this. As part of a clinical quality improvement initiative to assess barriers to success, we sought to increase the likelihood of data collection by leveraging an SMS-text messaging-based program on all discharged acute stroke patients for a several-month period. Using the services offered by a digital health technology company [Philips Patient Navigation Manager (formerly Medumo), Boston, MA, United States], we developed and launched an SMS-text follow-up program for patients discharged from the Massachusetts General Hospital (MGH) with a stroke ICD-10 code (I63, I60, I61, and G45) between June 8, 2020, and February 1, 2021. Patients were eligible to participate in the program if they had a valid mobile phone contact number in their medical chart. Patients who had not previously consented to receive SMS texts from their clinical care team at MGH received one consent SMS-text message at the time of acute hospital discharge, which remained active (i.e., giving the option to consent) for the duration of the program. If they did not consent, they did not receive any further messages. Patients had the option to decline participation in the program by responding “STOP” or simply not responding to the consent message.

Patients who consented to receiving SMS texts were enrolled in the program at the time of discharge regardless of their discharge destination (home or facility) and were provided instructions for unsubscribing (Supplementary Table S1). To familiarize patients with the SMS-texting method of communication and optimize patient engagement, patients also received weekly brain health educational tips developed by a multidisciplinary panel of clinicians, including neurologists, dietitians, and therapists (Supplementary Table S1).

Enrolled patients received an SMS text at 30, 60, and 90 days after hospital discharge, prompting them to complete the simplified modified Rankin scale (mRS) (20), a single-item, seven-level, ordinal measure of global disability, and then the Patient-Reported Outcomes Measurement Information System (PROMIS) Global-10, a 10-item measure of physical health, mental health, social health, pain, fatigue, and overall perceived quality of life. Individual items from each assessment were sent *via* SMS-text messages one at a time (i.e., each question of each assessment was one text message). Participants responded by directly texting the number corresponding to the answer of their selection. Participants had 1 week from receiving the prompt to complete the assessment at each time point. Responses were automatically saved in a secure database. Participants who completed all questions associated with the mRS at any given time point were considered responders, while those that did not were considered nonresponders. Participants who completed the mRS but did not complete all questions associated with the PROMIS Global-10 were still considered responders but were not scored on this assessment. The mRS can be dichotomized into good (score 0–2) and poor (score 3–6) outcomes (21). Global Physical Health (GPH) and Global Mental Health (GMH) *z*-scores (mean: 50; standard deviation (SD): 10) were derived from two 4-item summary scores extracted from the PROMIS Global-10 questions (22). At the conclusion of the program, enrolled patients received a satisfaction survey *via* an SMS text with six questions that had multiple-choice response options. Participants were counted as responders to the satisfaction survey if they responded to at least one question.

To evaluate the reliability of outcome measure scores obtained *via* SMS texting, mRS and PROMIS Global-10 scores closest in time to SMS-text responses were extracted from documented traditional follow-up encounters (clinic visit or follow-up phone call), when available. The clinically documented score was compared to the score from the closest SMS-text response in time.

To compare the yield and cost of the SMS texting approach to gather outcomes after acute stroke discharge with traditional methods of ascertaining outcomes, we added clinical staff and utilized a trained coordinator to call all consecutive patients discharged with stroke during a 3-month period to obtain their outcomes approximately 90 days after hospitalization discharge. The mRS and PROMIS Global-10 scores were also assessed *via* phone calls in the same order as SMS texts. Three attempts were made to reach each patient. Call attempts and time lengths were documented. The results of this intervention were then used to compare the cost between the two strategies (SMS texts vs. phone calls) for gathering poststroke outcomes.

### Statistical analysis

Participant characteristics were examined with mean and SD, median and interquartile range (IQR), or *n* (%). Independent sample *t*-tests and chi-squared tests of independence were performed to compare clinical and demographic characteristics between those enrolled in the program and those not enrolled and between those who responded to the assessment prompts (responders) and those who did not (nonresponders).

Weighted Cohen’s kappa (23) was calculated to assess the agreement between SMS texts and clinician- or coordinator-gathered responses for the mRS, and polychoric correlations were calculated to assess the agreement between GPH and GMH scores (subscales of PROMIS Global-10). For the mRS, we examined agreement using the ordinal level (with quadratic weights, Supplementary Table S3) and the dichotomized level (good vs. poor) outcomes.

Descriptive statistics were used to characterize the time, yield, and cost comparison of SMS texting vs. phone call-based methods of gathering outcomes and to examine the results of the satisfaction survey.

The Massachusetts General Brigham Institutional Review Board (#2021P001342) approved this study, which was exempt from written informed consent as the data extracted for this study were gathered under the standard of care through a quality improvement initiative. Data will be made available from the corresponding author upon reasonable request.

## Results

**Figure 1:**
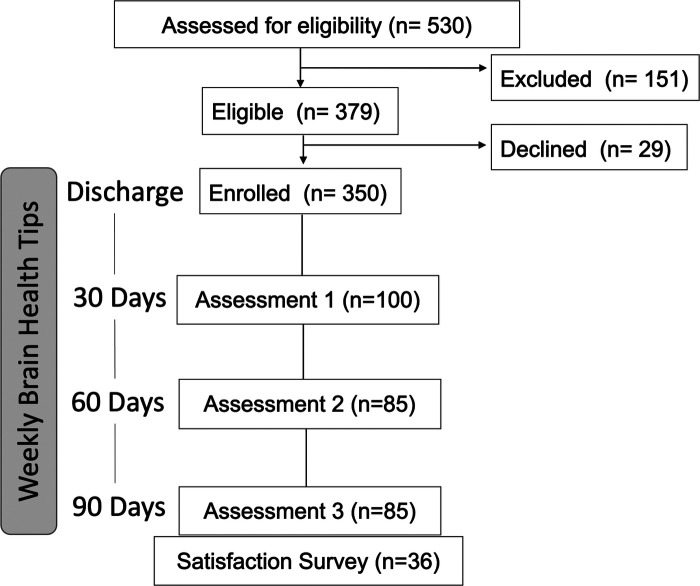
Study flow diagram. Cohorts at each assessment time point are unique.

Of the 530 patients discharged from MGH with a stroke ICD-10 code between June 8, 2020, and February 1, 2021, 350 patients (66.04%) were enrolled in the program. Patients enrolled on average 6.8 ± 8.2 (mean ± SD) days after acute stroke hospital admission. Patients were not enrolled if they did not have valid contact information in the medical chart (*n* = 151) or declined to receive messages (*n* = 29) (Figure 1). Enrolled patients were more likely to have English listed as their preferred language (*χ*^2^ = 5.44, *p* = 0.02), have a lower NIH stroke scale upon admission (*t* = 5.0, *p *≤ 0.001), and have been discharged directly home from the hospital (*χ*^2^ = 4.20, *p* = 0.04) compared to those patients who did not enroll (Table 1).

**Table 1:** Cohort demographic and clinical characteristics.

	Enrolled (350)	Not enrolled (180)	*p*	Responders (142)	Nonresponders (208)	*p*
Age	66.3 ± 16.8	69.4 ± 16.7	0.5	65.2 ± 15.1	67.0 ± 17.7	0.06
Sex (male)	196 (56.0%)	92 (51.1%)	0.3	78 (54.9%)	118 (56.7%)	0.6
Hospital LOS	6.8 ± 8.2	8.1 ± 7.6	0.3	5.5 ± 6.4	7.6 ± 9.1	0.01*
NIH stroke scale^a^	3 [1–6]	5 [2–11]	<0.001*	3 [1–6]	8 [5–14]	<0.001*
**Preferred language**
English	312 (89.1%)	154 (85.6%)	0.02*	133 (93.7%)	179 (86.1%)	0.009*
Other	38 (10.9%)	26 (14.4%)		9 (6.3%)	29 (13.9%)	
**Discharge destination**
Home	200 (57%)	86 (48%)	0.04*	100 (70.4%)	100 (48.1%)	<0.001*
Facility	150 (43%)	94 (52%)		42 (29.6%)	108 (51.9%)	
**Principal problem**
Ischemic	237 (67.7%)	124 (68.5%)	0.2	94 (66.2%)	143 (68.8%)	0.5
Hemorrhagic	90 (25.7%)	51 (28.3%)		37 (26.1%)	53 (25.5)	
TIA	23 (6.6%)	5 (2.8%)		11 (7.8%)	12 (5.8)	

LOS, length of stay; TIA, transient ischemic attack.

Scores reported as mean ± SD, median [interquartile range], or n (%).

*denotes statistical significance.

^a^ NIH stroke scale scores at acute stroke hospital admission were available for 241/350 (68.9%) enrolled, 127/180 (70.6%) not enrolled, 100/142 (70.4%) responders, and 141/208 (67.8%) nonresponders.

**Figure 2:**
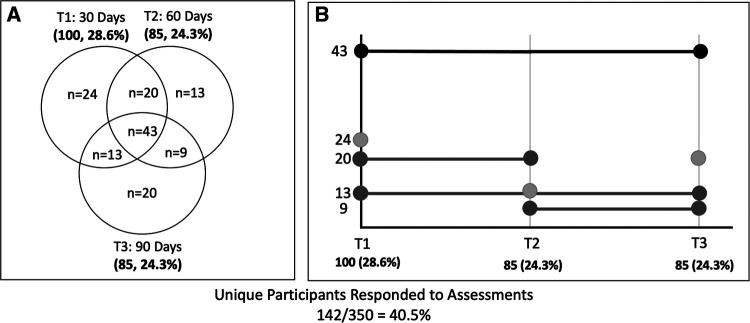
(**A**) Number of SMS-text assessment responses at different time points depicted in a Venn diagram (*N*, *N*%). *N* indicates unique participant responses. For example, *N* = 13 participants responded at 60 days poststroke, *N* = 20 answered at both 30 and 60 days, and *N* = 43 responded at all three study time points. (**B**) SMS-text assessment compliance over time. Shades of gray (dark to light) correspond to those that responded at all time points, those that responded at two timepoints (T_1_ and T_2_, T_1_ and T_3_, or T_2_ and T_3_), and those that responded at a single time point (T_1_, T_2_, or T_3_). SMS, short message service.

Of those who enrolled, 40.5% (*n* = 142) responded to at least one SMS text to complete an assessment. The response rate at 30-day postdischarge was 28.6%, and the response rates at the 60- and 90-day time points were 24.3%. Of the responders, 30% (*n* = 42) responded at any two time points and 30% (*n* = 43) of responders responded at all three time points (Figure 2A). SMS-text response compliance is presented in Figure 2B. Responders to message prompts were more likely to have English as their preferred language (*χ*^2^ = 9.33, *p* = 0.009), have shorter acute hospital length of stay (*t* = 2.5, *p* = 0.01), have a lower NIH stroke scale upon admission (*t* = 3.98, *p* < 0.001), and have been discharged directly home (*χ*^2^ = 24.94, *p* < 0.001) (Table 1).

**Figure 3:**
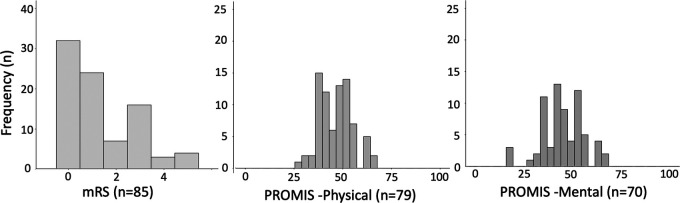
Distributions of 90-day outcomes (mRS, PROMIS Physical, and PROMIS Mental) gathered *via* SMS texting. SMS, short message service; mRS, modified Rankin scale; PROMIS Physical, Global Physical Health Score; PROMIS Mental, Global Mental Health Score.

The median (IQR) modified Rankin scale score collected by SMS testing was 1 (0–3) at 30-, 60-, and 90-day postdischarge. The median (IQR) GPH scores were 44.9 (42.3–50.8), 47.7 (42.3–54.1), and 47.7 (41.1–54.1) and the median GMH scores were 45.8 (38.8–50.8), 43.5 (38.8–53.3), and 45.8 (36.9–50.8) at 30-, 60-, and 90-day postdischarge, respectively (Supplementary Table S2). The distributions of these outcomes gathered *via* SMS texting at 90 days are shown in Figure 3.

**Figure 4:**
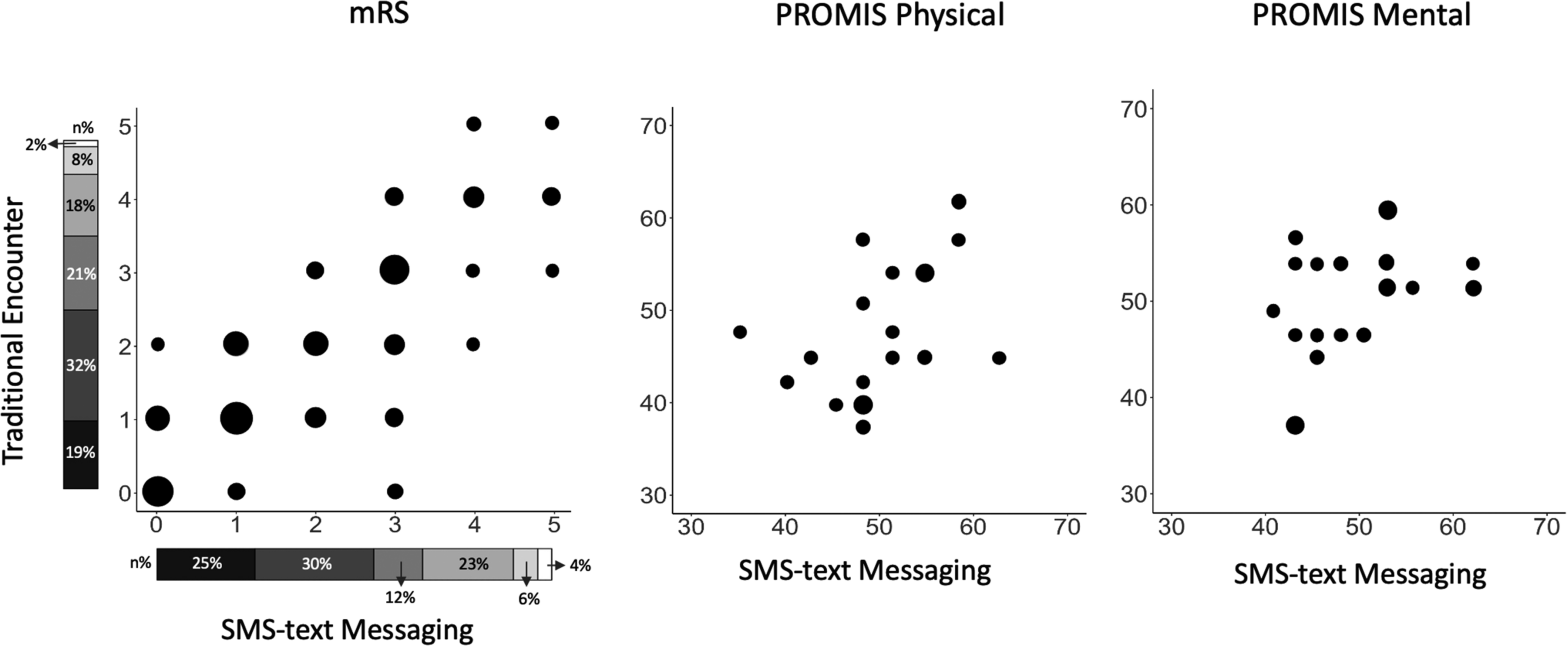
(**A**) mRS compared by modality (traditional encounter vs. SMS texting) across all collection time points (30, 60, and 90 days) for *N* = 113. Bubble plot to assess agreement between outcomes collected by traditional programs vs. SMS texting. Sizes of bubbles are directly proportional to the number of participants who provided answers *via* traditional methods and SMS texting. Subjects with data at multiple time points were compared at the latest available time point. The stacked bar near axes shows mRS distributions as collected by each modality with dark to light gradient representing scores 0–5, respectively. (**B**) PROMIS Physical and PROMIS Mental compared by modality for *N* = 19. Bubble plot to assess agreement between outcomes collected by traditional programs vs. SMS texting. The size of bubbles is directly proportional to the number of participants who provided answers *via* traditional methods and SMS texting. SMS, short message service; mRS, modified Rankin scale; PROMIS Physical, Global Physical Health Score; PROMIS Mental, Global Mental Health Score.

The mRS [median: 1 IQR: 1–3) from clinical follow-up encounters, within 13.0 ± 14.9 days of SMS-text responses across collection time points (Supplementary Figure S1), was available for 113 of the 142 patients who responded (Figure 4). Weighted Cohen’s kappa between mRS scores obtained from SMS texting compared to follow-up encounters revealed almost perfect agreement (κ = 0.8, *p* < 0.001) for dichotomized (good vs. poor) and ordinal (with quadratic weights, Supplementary Table S3) levels (κ = 0.8, *p* < 0.001) of the mRS. The PROMIS Global-10 score was not routinely collected and so was only available for 19 patients from clinical encounters. There were significant associations between traditional methods and SMS texting of ascertaining PROMIS subscores (ρ = 0.4, *p* < 0.01, GPH, and ρ = 0.4, *p* < 0.01, GMH) (Figure 4).

To compare the yield of SMS texts with that of phone calls, we attempted to complete a 90-day phone call for all patients discharged within a 3-month time period. Of the 169 stroke patients discharged, we reached 104 (61.4%) by phone. Of those reached by phone, 59 (56.7%) were contacted on the first call attempt, 28 (26.9%) were contacted on the second attempt, and 17 (10.1%) were contacted on the third attempt. For every successful phone call, there were 2.5 unanswered calls. As compared to those who did not answer, patients who answered the phone calls were more likely to have English listed as their preferred language (*t* = 3.9, *p *≤ 0.001). Phone calls took 5.4 ± 2.9 minutes to complete. Unanswered calls took approximately 1.5 minutes.

We estimated the cost per successful assessment achieved *via* SMS texts vs. phone calls. The total annual cost of the SMS-text program was $12,500 to configure and run, with a cost per assessment defined as $\frac{\$12,500}{a}$ (where *a* = # of assessments). For phone calls, we estimated a fixed cost of 20% of a coordinator’s time (for training and documentation) and added the average cost per phone call (both successful and unsuccessful) to each patient (*n*). In Boston, Massachusetts, coordinators make an average (avg.) of $60,000 and thus $0.48 per minute. Answered phone calls cost $2.50 (5.4min×$0.48/min), while unanswered phone calls cost $0.70(1.5min×$0.48) on average. This analysis yielded the following:

$$\frac{\begin{array}{c} {}[\mathrm{fixed}\;\mathrm{coordinator}\;\mathrm{cost}+(\mathrm{avg}.\;\;\mathrm{cost}\;\mathrm{of}\;a\;\mathrm{successful}\;\mathrm{call}\times n)\\ +(\mathrm{avg}.\;\;\mathrm{cost}\;\mathrm{of}\;\mathrm{an}\;\mathrm{unsuccessful}\;\mathrm{call}\\ \times \mathrm{avg}.\;\mathrm{number}\;\mathrm{of}\;\mathrm{unanswered}\;\mathrm{calls}\times n)] \end{array}}{n}$$

or

$$\frac{\left[\$12,000+(2.5\times n+\$0.70\times 2.5\times n)\right]}{\mathrm{number}\;\mathrm{of}\;\mathrm{subjects}},$$

suggesting that SMS texts become less costly than traditional phone calls if used for outcomes assessment in more than 181 stroke patients per year.

The results of the satisfaction survey revealed that the majority of patients found the educational material received *via* SMS texts to be helpful (53%), with only a small portion of patients finding information burdensome (6%). Most participants felt there was just the right number of tips (79%) and that the messages were clear and easy to understand (97%). Most felt that they were able to easily pick between the choices (91%) in the SMS texts that best described their recovery. Most participants (91%) reported feeling as comfortable answering questions by SMS texts than by answering in person or *via* phone calls (Table 2).

**Table 2:** Satisfaction survey results.

Question	*n* (%)
**1: How helpful was it to receive a text with information, educational content, and tips about stroke?**	
I did not find it helpful at all	2 (5.6)
Neutral	15 (41.7)
I found it very helpful	19 (52.7)
**2: Did you find receiving information about stroke *via* text burdensome?**	
I did not find the program burdensome	31 (93.9)
I found the program burdensome	2 (6.1)
**3: What did you think of the number of tips and questions?**	
Too few reminders	1 (3.0)
Just the right number of reminders	26 (78.8)
Too many reminders	6 (18.2)
**4: The text messages about stroke were clear and easy to understand.**	
Agree	32 (97.0)
Disagree	1 (3.0)
**5: I was able to easily pick between the choices in the text messages that best described my degree of stroke recovery.**	
Agree	30 (90.9)
Disagree	3 (9.1)
**6: I was as comfortable answering questions by text as if I were answering in person or on the phone.**	
Agree	30 (90.9)
Disagree	3 (9.1)

Satisfaction survey results are reported at n (%).

## Discussion

This is the first study to systematically examine the feasibility, reliability, cost, and acceptability of gathering outcomes after acute stroke *via* SMS texts. This novel method was found to be highly reliable for collecting the mRS scores and moderately reliable for collecting PROM scores. Even without specific program marketing or patient engagement campaigns, the SMS-text program yielded a 40.5% response rate. Compared to direct patient phone calls, SMS texting yielded fewer responses but is cost-saving for centers with annual stroke discharges exceeding 181 patients based on our costing equation. The experience of receiving text messages with assessments and brain health tips was overall very well received by stroke patients.

We found almost perfect agreement between mRS scores at both the dichotomized (good vs. poor outcome) and ordinal levels obtained *via* SMS texting compared to traditional methods. This high reliability provides the foundation for systematic evaluation of stroke survivors’ outcomes at a large scale, which could help evaluate the efficacy of stroke treatments. We found moderate agreement with traditional methods for patient-reported measures. This suggests that certain stroke outcomes (i.e., ordinal ratings of global disability) may be more suitable for text-message programs. Prior studies for smoking cessation (24) and depression (25) have also found mixed reliability (fair to substantial) for self-report assessments. Digital means for collecting stroke outcomes may be limited by stroke-related impairments (i.e., language or cognitive deficits). Further exploration is required to examine the reliability of collecting different types of outcomes *via* SMS texting, particularly in stroke. Furthermore, modality-specific outcome measures, such as motor or language assessments after stroke, may require other types of digital or sensor-based approaches (26–28).

SMS texting had an overall lower yield (40.5%) in this study compared to targeted phone call follow-up method (61%, phone calls). The first attempt at reaching participants *via* SMS texts also yielded lower responses (28.6%) than the first attempt at reaching participants *via* phone calls (56.7%). This differs from a prior study that received more SMS-text data than paper diary data after birth control insertion, although notably this patient population was substantially younger as compared to ours (29). Stroke survivors who tend to be older and often suffer stroke-related deficits may be limited in their ability to use cell phones or read and write SMS texts. Survivors might also have limited access to their mobile devices when discharged to a facility, as a majority (70.4%) of responders were discharged home. Engaging caregivers to help with outcomes data collection *via* digital technology may be helpful in these cases. Low SMS-text response rates could also be attributed to participants feeling more comfortable declining to participate *via* SMS texts rather than directly to a care team member *via* phone calls. Another reason could be that the number of required questions needed to complete the assessments by SMS texts was too burdensome, and future research should determine the optimal number of questions to response ratio. In addition, future programs with a dedicated patient and caregiver outreach and SMS-text reminders (30) will likely yield higher response rates.

While the majority of stroke patients consented to SMS-text communication, there were differences between those who consented and those who did not. Individuals who consented to receive SMS texts were more likely to have English listed as their preferred language than another language, have a lower NIH stroke scale at admission, and be discharged home rather than to a postacute care facility. Similar differences were found between those who responded to SMS-text assessments vs. those who did not. Furthermore, outcomes gathered *via* SMS texts revealed that responders had predominantly mild disability (median mRS of 1, with an interquartile range of 0-3). Outcomes gathered *via* SMS texts may not be representative of all stroke survivors. A nutrition education program for low-income parents that used SMS texts for program evaluation also found limitations in those who could be reached (31). In stroke, different approaches may be required to reach non-English speakers (32) and those with more severe disabilities (33). If the use of SMS-text programs can diminish the burden of manual collection, outcome collection systems can work in parallel to focus human resources on the more healthcare marginalized and disabled.

Due to the fixed cost of the SMS-text program for an unlimited number of participants, higher response rates would render a lower cost per participant. This contrasts with traditional methods such that outcomes collected during clinic visits or by phone calls require more resources for additional participants and thus cost increases per participant. Therefore, identifying the cost-to-response ratio that would favor SMS texting over traditional methods is essential for developing cost-saving programs. In our study, we show that gathering data *via* SMS texting would be cost-saving at scale for larger populations. Alternatively, it could also be cost-effective for smaller populations requiring outcome assessments at a greater frequency. For example, a previous study showed that SMS texting was more cost-effective for a weekly, two-question survey than the same paper-based survey (34) due to the frequency demand of the assessment. Future programs should consider the number of participants, data type, and sampling frequency and length in determining the cost-to-benefit ratio when using SMS texting.

Patients who participated in the program found it acceptable without additional burden. The majority found that the messages were clear, easy to understand, and easy to answer. The delivery of brain health tips was well received. These results are consistent with results from the acceptability of SMS texting in diverse clinical populations including individuals with depression (25), high blood pressure (35), and psychosis (36). Given the pervasive use of cell phones in modern society, cell phone-based outcome programs have significant promise for a wide range of patient populations including those with stroke. Although it is feasible to collect the mRS score *via* a mobile app (19), SMS texting leverages existing software without requiring a smartphone, additional download, or application knowledge.

Overall, our findings suggest that collecting functional outcomes *via* SMS texting during the first 3 months after stroke is feasible, acceptable, and reliable but that reach and cost-effectiveness should be further considered for broad clinical translation. Future programs should consider developing content in multiple languages and incorporating dedicated patient outreach materials. For example, educational material on how to view and respond to SMS-text communications delivered during the acute stroke inpatient stay could be helpful. Such content would help reach vulnerable populations. Increasing the yield of SMS-text outcome programs would decrease the cost per participant and make broad adoption across healthcare systems more feasible.

This study has several important limitations. The study was conducted at a single, urban, academic medical center in the northeastern United States with a predominantly White patient population. Findings may not be translatable to other hospitals in different locations with different patient populations. A limitation to communication *via* SMS texting is the chance that someone other than the intended recipient is receiving or interacting with the SMS texts. Moreover, the program could not gather information on the number of subjects who passed away during the study. In longitudinal data collection *via* SMS texting, subjects may see their responses from prior time points (in the SMS-text chain), which may lead to recall bias. Our sample of PROM data *via* both SMS texting and traditional methods was small (*n* = 19), and thus future studies with larger samples are needed to draw definitive conclusions. At last, the potential effects of our stroke and brain health educational program delivered *via* SMS texting were not systematically considered in our cost analysis.

## Conclusion

Our study suggests that it is feasible, reliable, and acceptable to provide general stroke education and gather functional outcome measures *via* SMS-text messaging after acute stroke discharge. Replication of our results in an independent cohort and further validation of specific outcome types and assessment frequency and length are warranted. Our findings lay the foundation for using SMS texting to gather outcomes after stroke to better evaluate the real-world efficacy of stroke therapies.

## Data Availability

The raw data supporting the conclusions of this article will be made available by the authors, without undue reservation.

## References

[1] Virani SS Alonso A Aparicio HJ Benjamin EJ Bittencourt MS Callaway CW Heart disease and stroke statistics—2021 update. Circulation. (2021) 143:e254–743. 10.1161/CIR.0000000000000950 33501848 PMC13036842

[2] Friedman HS. Tissue plasminogen activator for acute ischemic stroke. N Engl J Med. (1996) 334:1405. 10.1056/NEJM199605233342114 8614437

[3] Goyal M Demchuk AM Menon BK Eesa M Rempel JL Thornton J Randomized assessment of rapid endovascular treatment of ischemic stroke. N Engl J Med. (2015) 372:1019–30. 10.1056/NEJMoa1414905 25671798

[4] Dawson J Liu CY Francisco GE Cramer SC Wolf SL Dixit A Vagus nerve stimulation paired with rehabilitation for upper limb motor function after ischaemic stroke (VNS-REHAB): a randomised, blinded, pivotal, device trial. Lancet. (2021) 397:1545–53. 10.1016/S0140-6736(21)00475-X 33894832 PMC8862193

[5] Dromerick AW Geed S Barth J Brady K Giannetti ML Mitchell A Critical period after stroke study (CPASS): a phase II clinical trial testing an optimal time for motor recovery after stroke in humans. Proc Natl Acad Sci U S A. (2021) 118:e2026676118. 10.1073/pnas.2026676118 34544853 PMC8488696

[6] Chiu C-C Wang J-J Hung C-M Lin H-F Hsien H-H Hung K-W Impact of multidisciplinary stroke post-acute care on cost and functional status: a prospective study based on propensity score matching. Brain Sci. (2021) 11:161. 10.3390/brainsci11020161 33530541 PMC7912561

[7] Tyagi S Koh GC-H Luo N Tan KB Hoenig H Matchar DB Role of caregiver factors in outpatient medical follow-up post-stroke: observational study in Singapore. BMC Fam Pract. (2021) 22:74. 10.1186/s12875-021-01405-z 33853544 PMC8048235

[8] Koonin LM Hoots B Tsang CA Trends in the use of telehealth during the emergence of the COVID-19 pandemic—United States, January–March 2020. Morb Mortal Wkly Rep. (2020) 69:1595–9. 10.15585/mmwr.mm6943a3 PMC7641006 33119561

[9] Naito A Wills A-M Tropea TF Ramirez-Zamora A Hauser RA Martino D Expediting telehealth use in clinical research studies: recommendations for overcoming barriers in North America. NPJ Parkinsons Dis. (2021) 7:34. 10.1038/s41531-021-00177-8 33846349 PMC8041858

[10] Bhavnani SP Narula J Sengupta PP. Mobile technology and the digitization of healthcare. Eur Heart J. (2016) 37:1428–38. 10.1093/eurheartj/ehv770 26873093 PMC4914890

[11] Pew-Research-Center. Mobile phone ownership over time. Mobile fact sheet (2021). Available from: https://www.pewresearch.org/internet/fact-sheet/mobile/

[12] Schwamm LH. Telehealth: seven strategies to successfully implement disruptive technology and transform health care. Health Aff. (2014) 33:200–6. 10.1377/hlthaff.2013.1021 24493761

[13] Iribarren S Beck S Pearce PF Chirico C Etchevarria M Cardinale D TextTB: a mixed method pilot study evaluating acceptance, feasibility, and exploring initial efficacy of a text messaging intervention to support TB treatment adherence. Tuberc Res Treat. (2013) 2013:349394. 24455238 10.1155/2013/349394 PMC3876704

[14] Rathbone AL Prescott J. The use of mobile apps and SMS messaging as physical and mental health interventions: systematic review. J Med Internet Res. (2017) 19:e295. 10.2196/jmir.7740 28838887 PMC5590007

[15] Mougalian SS Gross CP Hall EK. Text messaging in oncology: a review of the landscape. JCO Clin Cancer Inform. (2018) 2:1–9. 10.1200/CCI.17.00162 30652579

[16] Bobrow K Brennan T Springer D Levitt NS Rayner B Namane M Efficacy of a text messaging (SMS) based intervention for adults with hypertension: protocol for the star (SMS text-message adherence support trial) randomised controlled trial. BMC Public Health. (2014) 14:28. 10.1186/1471-2458-14-28 24410738 PMC3909351

[17] Naughton F Prevost AT Gilbert H Sutton S. Randomized controlled trial evaluation of a tailored leaflet and SMS text message self-help intervention for pregnant smokers (MiQuit). Nicotine Tob Res. (2012) 14:569–77. 10.1093/ntr/ntr254 22311960

[18] Keoleian V Polcin D Galloway GP. Text messaging for addiction: a review. J Psychoact Drugs. (2015) 47:158–76. 10.1080/02791072.2015.1009200 PMC4537651 25950596

[19] Cooray C Matusevicius M Wahlgren N Ahmed N. Mobile phone–based questionnaire for assessing 3 months modified Rankin score after acute stroke. Circ Cardiovasc Qual Outcomes. (2015) 8:S125–30. 10.1161/CIRCOUTCOMES.115.002055 26515200

[20] Bruno A Akinwuntan AE Lin C Close B Davis K Baute V Simplified modified Rankin scale questionnaire. Stroke. (2011) 42:2276–9. 10.1161/STROKEAHA.111.613273 21680905

[21] Sulter G Steen C De Keyser J. Use of the Barthel index and modified Rankin scale in acute stroke trials. Stroke. (1999) 30:1538–41. 10.1161/01.STR.30.8.1538 10436097

[22] Hays RD Bjorner JB Revicki DA Spritzer KL Cella D. Development of physical and mental health summary scores from the patient-reported outcomes measurement information system (PROMIS) global items. Qual Life Res. (2009) 18:873–80. 10.1007/s11136-009-9496-9 19543809 PMC2724630

[23] Warrens MJ. Cohen’s weighted kappa with additive weights. Adv Data Anal Classif. (2013) 7:41–55. 10.1007/s11634-013-0123-9

[24] Thrul J Mendel JA Simmens SJ Abroms LC. Collecting outcome data of a text messaging smoking cessation intervention with in-program text assessments: how reliable are the results? Addict Behav. (2018) 85:31–7. 10.1016/j.addbeh.2018.05.012 29807305 PMC6015545

[25] Richmond SJ Keding A Hover M Gabe R Cross B Torgerson D Feasibility, acceptability and validity of SMS text messaging for measuring change in depression during a randomised controlled trial. BMC Psychiatry. (2015) 15:68. 10.1186/s12888-015-0456-3 25886377 PMC4391083

[26] Cramer SC Koroshetz WJ Finklestein SP. The case for modality-specific outcome measures in clinical trials of stroke recovery-promoting agents. Stroke. (2007) 38:1393–5. 10.1161/01.STR.0000260087.67462.80 17332455

[27] Erler KS Wu R DiCarlo JA Petrilli MF Gochyyev P Hochberg LR Association of modified Rankin scale with recovery phenotypes in patients with upper extremity weakness after stroke. Neurology. (2022) 98(18):e1877–85. 10.1212/WNL.0000000000200154 35277444 PMC9109148

[28] Kim GJ Parnandi A Eva S Schambra H. The use of wearable sensors to assess and treat the upper extremity after stroke: a scoping review. Disabil Rehabil. (2021) 44:6119–38. 10.1080/09638288.2021.1957027 34328803 PMC9912423

[29] Nippita S Oviedo JD Velasco MG Westhoff CL Davis AR Castaño PM. A randomized controlled trial of daily text messages versus monthly paper diaries to collect bleeding data after intrauterine device insertion. Contraception. (2015) 92:578–84. 10.1016/j.contraception.2015.09.004 26363433 PMC4967589

[30] Moran L O’Loughlin K Kelly BD. The effect of SMS (text message) reminders on attendance at a community adult mental health service clinic: do SMS reminders really increase attendance? Ir J Med Sci. (2018) 187:561–4. 10.1007/s11845-017-1710-0 29143910

[31] Grutzmacher SK Munger AL Speirs KE Zemeir LA Richard KC Worthington L. Feasibility of bidirectional text messages in evaluating a text-based nutrition education program for low-income parents: results from the text2bhealthy program. Eval Program Plann. (2017) 64:90–4. 10.1016/j.evalprogplan.2017.04.001 28578291

[32] Al Shamsi H Almutairi AG Al Mashrafi S Al Kalbani T. Implications of language barriers for healthcare: a systematic review. Oman Med J. (2020) 35:e122. 10.5001/omj.2020.40 32411417 PMC7201401

[33] Lezzoni L. Eliminating health and health care disparities among the growing population of people with disabilities. Health Aff. (2011) 30:1947–54. 10.1377/hlthaff.2011.0613 21976339

[34] Johansen B Wedderkopp N. Comparison between data obtained through real-time data capture by SMS and a retrospective telephone interview. Chiropr Osteopat. (2010) 18:10. 10.1186/1746-1340-18-10 20500900 PMC2883994

[35] Leon N Surender R Bobrow K Muller J Farmer A. Improving treatment adherence for blood pressure lowering via mobile phone SMS-messages in South Africa: a qualitative evaluation of the SMS-text adherence support (STAR) trial. BMC Fam Pract. (2015) 16:80. 10.1186/s12875-015-0289-7 26137844 PMC4490665

[36] D'Arcey J Collaton J Kozloff N Voineskos AN Kidd SA Foussias G. The use of text messaging to improve clinical engagement for individuals with psychosis: systematic review. JMIR Ment Health. (2020) 7:e16993. 10.2196/16993 32238334 PMC7163420

