# Supplementary material for: SMS-text messaging for collecting outcome measures after acute stroke
Source: Front Digit Health. 2023 Feb 23;5:1043806. doi: 10.3389/fdgth.2023.1043806 (PMC9996089; doi:10.3389/fdgth.2023.1043806)
Supplement: Supplementary file 1 [file Table1.docx]

Supplementary Material

# Supplemental Table 1. Program Messages

| **Introduction Message** |
| --- |
| Hi, this is Mass General Neurology and Neurosurgery. We want to know how you are recovering after your recent admission. We will send you brain health tips and surveys over the next 12 weeks. (Msg rates may apply. Reply STOP to stop msgs) |
| **Consent Message (for those not already consented via medical chart)** |
| Reply “Y” to receive info related to your healthcare. As with any text, msgs may not be secure. Txt STOP to opt out. Msg&DataRatesMayApply |
| **Brain Health Education Tips** |
| Get active! Exercise 30 minutes a day keeps you healthier physically and mentally. Try taking a daily walk or taking the stairs instead of the elevator. |
| Can you recognize signs of a stroke? Remember FAST (Face, Arms, Speech, Time): if your face or arm is weak or your speech is off, call 9-1-1 immediately. |
| If you’re a smoker or vaper, it’s never too late to quit. Breaking the habit is hard. Ask your doctor for advice about quit-smoking aids like gum or patches. |
| Try a turkey burger instead of beef. Turkey, chicken, and fish are all lower in cholesterol. Less cholesterol means less plaque in your arteries. |
| Say no to sugar. Replace pastry or cake with fresh fruit. Just as sweet and may lower your risk for diabetes. |
| Feeling tired or fatigued after a stroke or brain injury is common. Keep at it! It will get better! |
| Feeling sad or down? You are not alone. Talk to your family, friends, or healthcare providers. We are all here to help. |
| It’s hard to remember to take different pills at different times. Organize a weekly pillbox or set a reminder on your phone. |
| Every day is a new chance to get stronger and to live healthier. |
| Small steps turn into miles. Your efforts today are moving you further on the path of recovery. |
| Celebrate your victories, big and small. Recovery after stroke or brain injury can be frustrating. Stay determined! |
| Keep connected with friends and family. They are on your shared road to recovery! |
| What’s your blood pressure? We want the top number to be less than 120. Check it at home and record them in a journal to discuss them with your doctor. |
| Practice the exercises you learned in therapy. They will keep you active, strengthen your muscles and increase your independence. Small changes, big rewards! |
| Feeling salty? Salt in your diet increases blood pressure and is hidden in many prepared foods: pizza, breads, canned foods, and soups. |
| Listen to your body. Challenge yourself but not beyond your limits. |

**Table 2**. Outcome Measure Scores Gather Via SMS-Texting

|  | **30 Days** | **60 Days** | **90 Days** |
| --- | --- | --- | --- |
| **mRS**  (n=100, 85, 85) | 1 [0-3] | 1 [0-3] | 1 [0-3] |
| **Global Physical Health**  (n=90, 70, 79) | 44.9 [42.3-50.8] | 47.7 [42.3-54.1] | 47.7 [41.05-54.01] |
| **Global Mental Health**  (n=90, 70, 70) | 45.8 [38.8-50.8] | 43.5 [38.8-53.3] | 45.8 [36.93-50.8] |

*Scores reported as Median [Interquartile Range]. mRS scores range from normal to bedridden (0-5). GPH z-scores range from poor to good, reported physical health (12.3-67.7) and GMH z-scores range from poor to good, reported mental health (21.2-67.6).*

**Table 3**. **Quadratic Weights Matrix: SMS-text Message verse Traditional Encounter**

|  |  |  | Rater 2 |  |  |  |
| --- | --- | --- | --- | --- | --- | --- |
| Rater 1 | 0 | 1 | 2 | 3 | 4 | 5 |
| 0 | 1 |  |  |  |  |  |
| 1 | 0.96 | 1 |  |  |  |  |
| 2 | 0.84 | 0.96 | 1 |  |  |  |
| 3 | 0.64 | 0.84 | 0.96 | 1 |  |  |
| 4 | 0.36 | 0.64 | 0.84 | 0.96 | 1 |  |
| 5 | 0 | 0.36 | 0.64 | 0.84 | 0.96 | 1 |

*Quadratic Weights matrix displays agreement between data collected via SMS-text message verse traditional encounters on the ordinal (0-5) mRS scale.*

**Figure 1**. **Distribution of Time Between Data Collection via SMS-Text and Traditional Encounter**


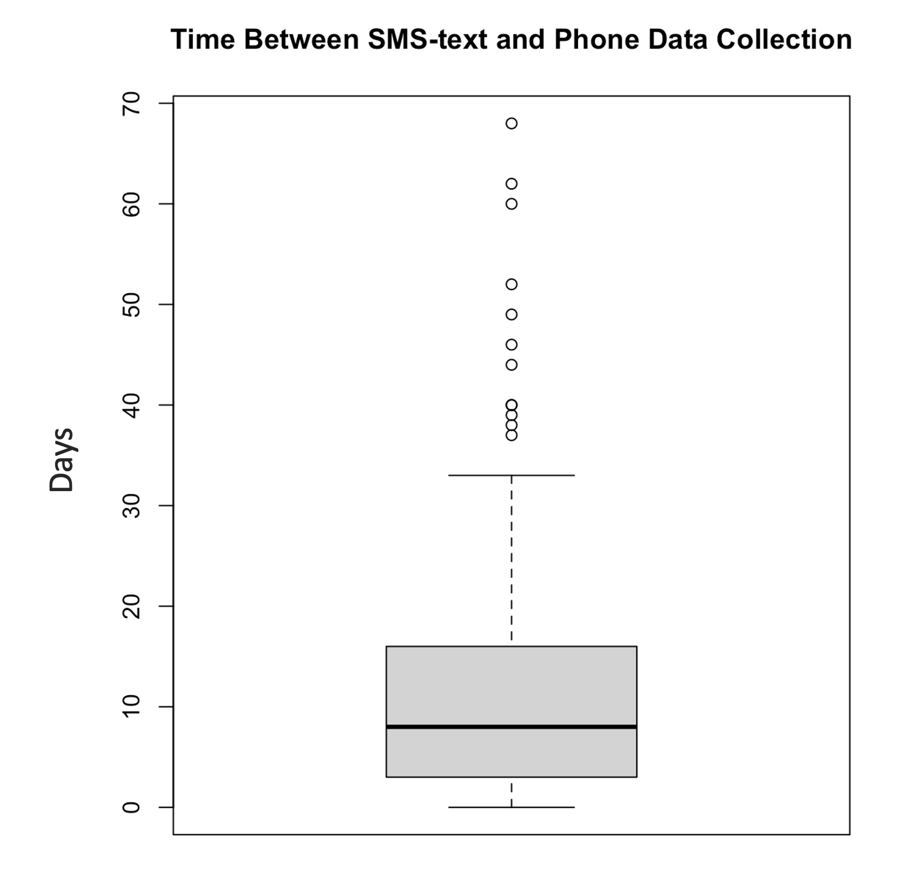


*Distribution of the time between when participants responded to an assessment survey via SMS-text message and when the assessment score was obtained during a traditional clinical encounter.*
